# Cracking the Code of Oscillatory Activity

**DOI:** 10.1371/journal.pbio.1001064

**Published:** 2011-05-17

**Authors:** Philippe G. Schyns, Gregor Thut, Joachim Gross

**Affiliations:** Institute of Neuroscience and Psychology, University of Glasgow, Glasgow, United Kingdom; Bremen University, Germany

## Abstract

Neural oscillations are ubiquitous measurements of cognitive processes and dynamic routing and gating of information. The fundamental and so far unresolved problem for neuroscience remains to understand how oscillatory activity in the brain codes information for human cognition. In a biologically relevant cognitive task, we instructed six human observers to categorize facial expressions of emotion while we measured the observers' EEG. We combined state-of-the-art stimulus control with statistical information theory analysis to quantify how the three parameters of oscillations (i.e., power, phase, and frequency) code the visual information relevant for behavior in a cognitive task. We make three points: First, we demonstrate that phase codes considerably more information (2.4 times) relating to the cognitive task than power. Second, we show that the conjunction of power and phase coding reflects detailed visual features relevant for behavioral response—that is, features of facial expressions predicted by behavior. Third, we demonstrate, in analogy to communication technology, that oscillatory frequencies in the brain multiplex the coding of visual features, increasing coding capacity. Together, our findings about the fundamental coding properties of neural oscillations will redirect the research agenda in neuroscience by establishing the differential role of frequency, phase, and amplitude in coding behaviorally relevant information in the brain.

## Author Summary

To recognize visual information rapidly, the brain must continuously code complex, high-dimensional information impinging on the retina, not all of which is relevant, because a low-dimensional code can be sufficient for both recognition and behavior (e.g. a fearful expression can be correctly recognized only from the wide-opened eyes). The oscillatory networks of the brain dynamically reduce the high-dimensional information into a low dimensional code, but it remains unclear which aspects of these oscillations produce the low dimensional code. Here, we measured the EEG of human observers while we presented them with samples of visual information from expressive faces (happy, sad, fear, etc.). Using statistical information theory, we extracted the low-dimensional code that is most informative for correct recognition of each expression (e.g. the opened mouth for “happy,” the wide opened eyes for “fear”). Next, we measured how the three parameters of brain oscillations (frequency, power and phase) code for low-dimensional features. Surprisingly, we find that phase codes 2.4 times more task information than power. We also show that the conjunction of power and phase sufficiently codes the low-dimensional facial features across brain oscillations. These findings offer a new way of thinking about the differential role of frequency, phase and amplitude in coding behaviorally relevant information in the brain.

## Introduction

Invasive and noninvasive studies in humans under physiological and pathological conditions converged on the suggestion that the amplitude and phase of neural oscillations implement cognitive processes such as sensory representations, attentional selection, and dynamical routing/gating of information [1]–[4]. Surprisingly, most studies have ignored how the temporal dynamics of phase code the sensory stimulus, focusing instead on amplitude envelopes (but see [5]), relations between amplitude and frequency [6], or coupling between frequencies ([7]–[10]; see [11] for a review). But there is compelling evidence that phase dynamics of neural oscillations are functionally relevant [12]–[16]. Furthermore, computational arguments suggest that if brain circuits performed efficient amplitude-to-phase conversion [17],[18], temporal phase coding could be advantageous in fundamental operations such as object representation and categorization by implementing efficient winner-takes-all algorithms [17], by providing robust sensory representations in unreliable environments, and by lending themselves to multiplexing, an efficient mechanism to increase coding capacity [18],[19]. To crack the code of oscillatory activity in human cognition, we must tease apart the relative contribution of frequency, amplitude, and phase to the coding of behaviorally relevant information.

We instructed six observers to categorize faces according to six basic expressions of emotion (“happy,” “fear,” “surprise,” “disgust,” “anger,” “sad,” plus “neutral”). We controlled visual information, by presenting on each trial a random sample of face information—smoothly sampled from the image using Gaussian apertures at different spatial frequency bands. The Gaussian apertures randomly sampled face parts simultaneously across the two dimensions of the image and the third dimension of spatial frequency bands (Figure S1 illustrates the sampling process for one illustrative trial; [20],[21]). We recorded the observers' categorization and EEG responses to these samples (see Materials and Methods, Procedure).

To quantify the relative coding properties of power, phase, and frequency, we used state-of-the-art information theoretic methods (Mutual Information, *MI*, which measures the mutual dependence between two variables; [22]) and computed three different *MI* measurements: between sampled pixel information and behavioral responses to each emotion category (correct versus incorrect), between EEG responses (for power, phase, and the conjunction of phase and power) and behavior, and finally between sampled pixel information and EEG response (see Figure S2 for the mutual information analysis framework and Computation: Mutual Information).

## Results

**Figure 1:**
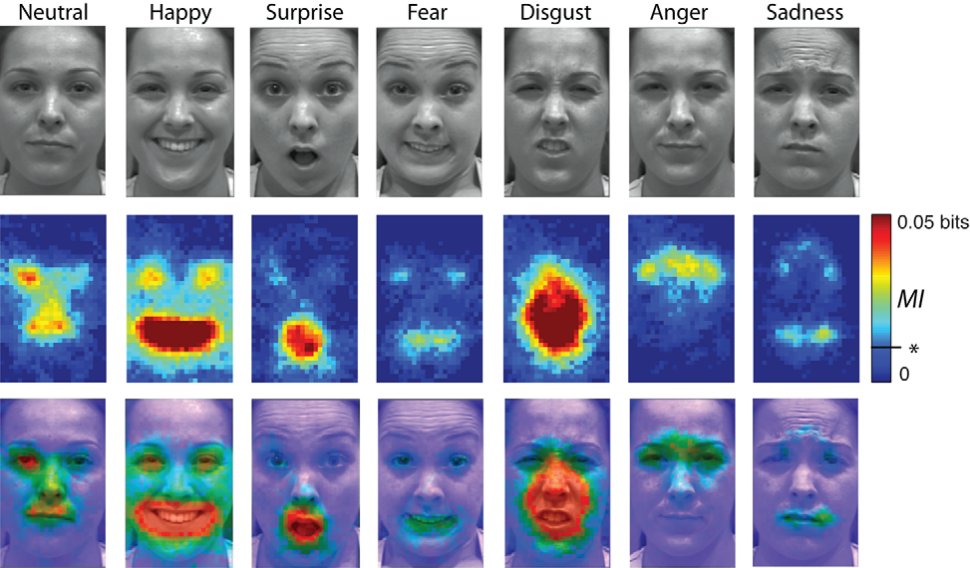
*MI*(Pixel; Behavior). The top rows of faces illustrate, from top to bottom, each expression of the experiment, the color-coded average *MI* (*n* = 6 observers) for each expression (*p*<.01 = .0094 bits, corrected, see * on the scale), an overlay of expression and *MI* for ease of feature interpretation.

First, to characterize the information that the brain processes in the cognitive task, for each observer and category, we computed *MI*(Pixel; Behavior), the *MI* between the distribution of grey-level values of each image pixel (arising from the summed Gaussian masks across spatial frequency bands, down-sampled from a 380×240 pixels image to a 38 to 24 image and gathered across trials) and equal numbers of correct versus incorrect categorization responses. Figure 1, *MI*(Pixel; Behavior) illustrates *MI* on a scale from 0 to 0.05 bits. High values indicate the face pixels (e.g., forming the mouth in “happy”) representing the visual information that the brain must process to correctly categorize the stimuli (see Figure S3 for a detailed example of the computation).

**Figure 2:**
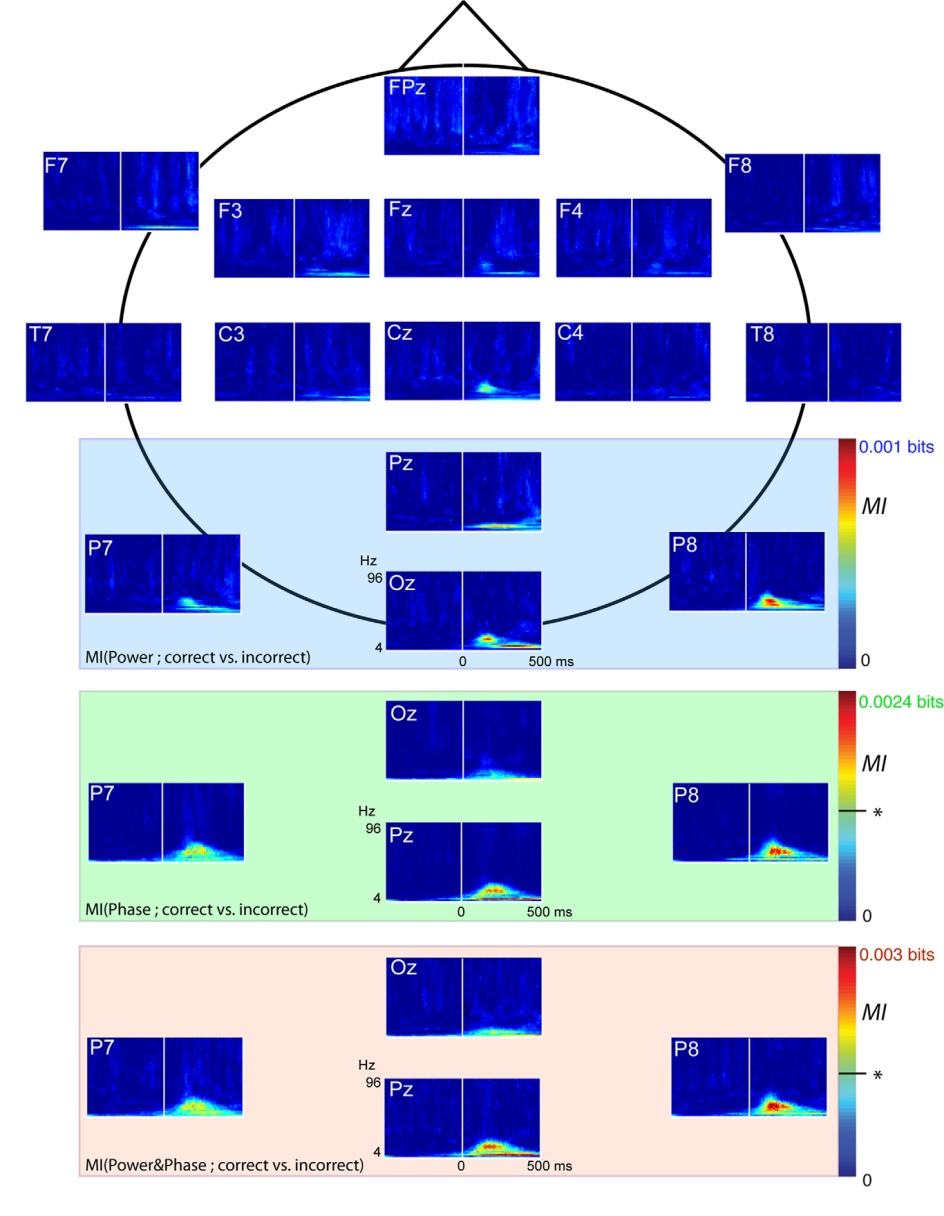
*MI*(EEG Response; Behavior). *MI* between behavior and the EEG average response for power, highlighted in the blue box for Pz, P8, P7, and Oz, phase (green box), and phase&power (red box), computed over the Time × Frequency space (*p*<.01  = .0013, see * on the scale).

We now compare how the parameters of oscillatory frequency, power, and phase code this information in the brain. For each observer, expression, electrode of the standard 10–20 position system, and trial, we performed a Time × Frequency decomposition of the signal sampled at 1,024 Hz, with a Morlet wavelet of size 5, between −500 and 500 ms around stimulus onset and every 2 Hz between 4 and 96 Hz. We make three points:

*(a) The conjunction of phase and power (phase&power) codes more information about complex categorization tasks than phase and power on their own.* In Figure 2, *MI*(EEG response; Behavior) measures the reduction of uncertainty of the brain response, when the behavioral variable correct versus incorrect categorization is known. We provide the measure for each electrode of the standard 10–20 position system over the Time × Frequency space. Pz, Oz, P8, and P7 had highest *MI* values of all electrodes, irrespective of whether the brain response considered was power (blue box), phase (green box), or the phase&power (red box). The adjacent *MI* scales reveal that phase&power was 1.25 times more informative of behavior than phase, itself 2.4 times more informative than power. Phase&power was 3 times more informative than power alone. Henceforth, the analyses focus on these four electrodes and on phase&power, the most informative brain measurement for the cognitive task.

**Figure 3:**
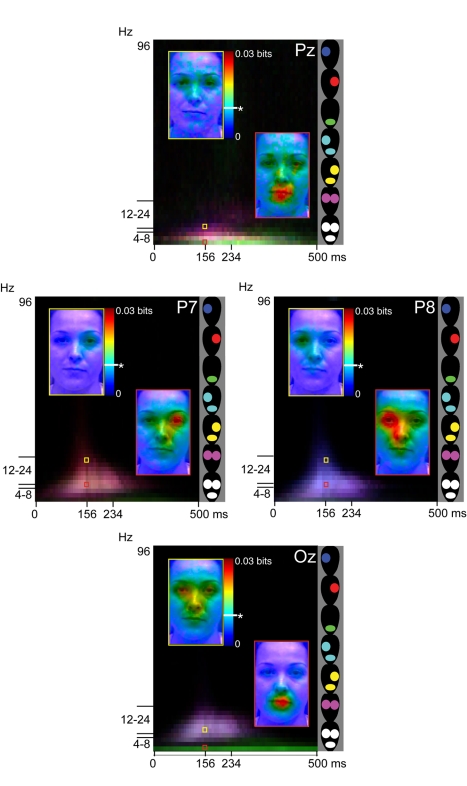
MI(Pixel; Phase&Power). For electrode Pz, P8, P7, and Oz, the color-coded pixels overlayed on a neutral face represent the average (*n* = 6) *MI* values for each face pixel and phase&power brain responses (see adjacent scale), at two different temporal frequencies (color-coded yellow and red), 156 ms following stimulus onset (*p*<.0000001 = .01 bits, uncorrected, see * on the scale). The underlying Time × Frequency space generalizes this analysis to each cell, using feature masks (left eye, mouth, right eye) and RGB coding to represent *MI* between combinations of these features (see adjacent schematic faces) and the phase&power EEG response. On Oz, the 4 Hz green strip illustrates high *MI* to the mouth, whereas the 8 to 24 Hz purple cloud represents *MI* to two eyes, indicating multiplexing of feature coding.

*(b) Phase&power codes detailed categorization-relevant features of sensory stimuli. MI*(Pixel; Behavior) revealed that the two eyes and the mouth are prominent features of expression discrimination (see Figure 1). As explained, with Gaussian masks we sampled pixels from the face on each trial. Consequently, for all correct trials of an expression category (e.g., “happy”), we can measure at each pixel location the mutual information between the distribution of grey-level values of the Gaussian masks across trials and each cell of the Time × Frequency brain response. Figure 3 reports *MI*(Pixel; Phase&Power), focusing on Pz, Oz, P8, and P7. The red box represents, at 4 Hz and 156 ms, following stimulus onset (a time point chosen for its prominence in face coding [21]), the color-coded *MI* value of each face pixel—overlayed on a neutral face background for ease of feature interpretation (the yellow box presents mutual information at 12 Hz and 156 ms). The scale is the adjacent rainbow colors ranging from 0 to 0.03 bits. Electrodes P7 (over left occipito-temporal cortex) and P8 (over right occipital-temporal cortex) reveal the highest *MI* to the contra-lateral eye (i.e., left eye for P8; right eye for P7). At the same time on Pz and Oz, the highest *MI* is to both eyes and to the mouth.

To generalize across Time × Frequency, for ease of presentation, we computed three masks extracting pixel locations from the left eye, right eye, and mouth. We averaged *MI* values within each mask, independently for each Time × Frequency cell. We then color-coded *MI* for each feature in RGB color space—red for “right eye,” green for “mouth,” and blue for “left eye”; see schematic colored faces adjacent to the Time × Frequency plot for complete color coding. The broad red (versus blue) cloud on electrode P7 (versus P8) denotes highest *MI* to the right (versus left) eye in this Time × Frequency region, whereas Pz and Oz demonstrate sensitivity to the two eyes (in purple) and to the mouth (in green). To conclude, phase&power codes detailed categorization-relevant features of the sensory input.

**Figure 4:**
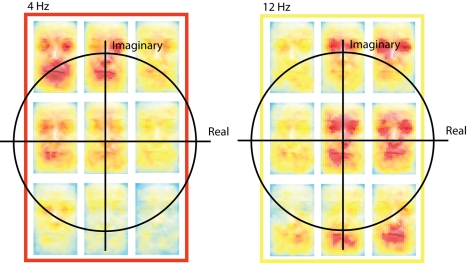
Mutual Information: The complex plane. For electrode Pz, the boxes develop the corresponding color-coded boxes in Figure 3. The red (4 Hz) and yellow (12 Hz) boxes represent the pixel mask values associated with a 3×3 discretization of the distribution of complex numbers. For each box, at 156 ms, for each correct trial we averaged the pixel values leading to this range of imaginary numbers—coded on an arbitrary scale between a low value of yellow (reflecting absence of this pixel in this range) and a high value of red (reflecting presence of this pixel in this range). The yellow box illustrates a phase&power coding of the two eyes (in red) between 45 and 90 deg of phase and a coding of the mouth (in red) between 270 and 315 deg of phase. The red box illustrates the coding of all three features (in red) between 90 and 135 deg of phase.

*(c) Phase&power coding is multiplexed across oscillatory frequencies.* Theta (4 Hz) and low beta (12 Hz) on both Oz and Pz demonstrate the remarkable multiplexing property of phase&power coding: the idea that the brain codes different information in different oscillatory bands. In Figure 3, Oz and Pz reveal that beta encodes two eyes (see the purple RGB code and the yellow framed faces) when theta encodes the mouth (see the green RGB code and the red framed faces). Multiplexing is also present to a lesser degree on P8 and P7. *MI* values critically depend on the joint distribution of variables (see Figure S3), and so we turn to Figure 4 to understand how the variables of phase and power jointly contribute to the coding of facial features. Figure 4 develops the red and yellow framed faces of Figure 3, for electrode Pz. At 156 ms, at 4 and 12 Hz, we discretized the distribution of power and phase neural responses in 3×3 bins—represented in Cartesian coordinates as 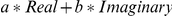. In each bin, we averaged the pixel values leading to this range of imaginary numbers. At 12 Hz, what emerges is a phase&power coding of the two eyes (in red, between 45 and 90 deg of phase) and an encoding of the mouth (in red, between 270 and 315 deg of phase). At 4 Hz, the encoding of mostly the mouth and the two eyes (in red) occurs between 90 and 135 deg of phase. The 4 and 12 Hz colored boxes in Figure 4 therefore illustrate the prominence of phase coding for facial features.

## Discussion

Here, using the concept of mutual information from Information Theory, we compared how the three parameters of neural oscillations (power, phase, and frequency) contribute to the coding of information in the biologically relevant cognitive task of categorizing facial expressions of emotion. We demonstrated that phase codes 2.4 times more information about the task than power. The conjunction of power and phase (itself 3 times more informative than power) codes specific expressive features across different oscillatory bands, a multiplexing that increases coding capacity in the brain.

In general, the relationship between our results on the frequency, power, and phase coding of neural oscillations cannot straightforwardly be related to the coding properties of more standard measures of the EEG such as event related potentials (ERP). However, an identical experimental protocol was run on the N170 face-sensitive potential [21],[23], but using reverse correlation analyses, not MI. Sensor analyses revealed that the N170 ERP initially coded the eye contra-lateral to the sensor considered, for all expressions, followed at the N170 peak by a coding of the behaviorally relevant information [21], together with a more detailed coding of features (i.e., with their Higher Spatial Frequencies) at the peak [23]. Interestingly, distance of behaviorally relevant information (e.g., the wide-opened eyes in “fearful” versus the mouth in “happy”) to the initially coded eye determined the latency of the N170 peak (with the ERP to a “happy” face peaking later than to a “fearful” face). ERPs confer the advantage of precise timing, leading to precise time course of coding in the brain, including phase differences across visual categories. However, we do not know whether this coding occurs over one or multiple sources of a network that might oscillate at different temporal frequencies (as suggested here between theta and beta), for example to code features at different spatial resolutions (as suggested in [19] and [24]). In sum, the complex relations between EEG/MEG data, the underlying cortical networks of sources, their oscillatory behaviors, and the coding of behaviorally relevant features at different spatial resolutions open a new range of fundamental questions. Resolving these questions will require integration of existing methods, as none of them is singly sufficient.

In these endeavors, the phase and frequency multiplexing coding properties of neural oscillations cannot be ignored.

## Materials and Methods

### Participants

Six observers from Glasgow University, UK, were paid to take part in the experiment. All had normal vision and gave informed consent prior to involvement. Glasgow University Faculty of Information and Mathematical Sciences Ethics Committee provided ethical approval.

### Stimuli

Original face stimuli were gray-scale images of five females and five males taken under standardized illumination, each displaying seven facial expressions. All 70 stimuli (normalized for the location of the nose and mouth) complied with the Facial Action Coding System (FACS, [25]) and form part of the California Facial Expressions (CAFE) database [26]. As facial information is represented at multiple spatial scales, on each trial we exposed the visual system to a random subset of Spatial Frequency (SF) information contained within the original face image. To this end, we first decomposed the original image into five non-overlapping SF bands of one octave each (120–60, 60–30, 30–15, 15–7.5, and 7.5–3.8 cycles/face, see Figure S1). To each SF band, we then applied a mask punctured with Gaussian apertures to sample SF face information with “bubbles.” These were positioned in random locations trial by trial, approximating a uniform sampling of all face regions across trials. The size of the apertures was adjusted for each SF band, so as to reveal six cycles per face. In addition, the probability of a bubble in each SF band was adjusted so as to maintain constant the total area of face revealed (standard deviations of the bubbles were 0.36, 0.7, 1.4, 2.9, and 5.1 cycles/degree of visual angle from the fine to the coarse SF band). Calibration of the sampling density (i.e., the number of bubbles) was performed online on a trial-by-trial basis to maintain observer's performance at 75% correct categorization independently for each expression. The stimulus presented on each trial comprised the randomly sampled information from each SF band summed together [27].

### Procedure

Prior to testing, observers learned to categorize the 70 original images into the seven expression categories. Upon achieving a 95% correct classification criterion of the original images, observers performed a total of 15 sessions of 1,400 trials (for a total of 21,000 trials) of the facial expressions categorization task (i.e., 3,000 trials per expression, happy, sad, fearful, angry, surprised, disgusted, and neutral faces, randomly distributed across sessions). Short breaks were permitted every 100 trials of the experiment.

In each trial a 500 ms fixation cross (spanning 0.4° of visual angle) was immediately followed by the sampled face information, as described before (see Figure S1). Stimuli were presented on a light gray background in the centre of a monitor; a chin-rest maintained a fixed viewing distance of 1 m (visual angle 5.36°×3.7° forehead to base of chin). Stimuli remained on screen until response. Observers were asked to respond as quickly and accurately as possible by pressing expression-specific response keys (seven in total) on a computer keyboard.

### EEG Recording

We recorded scalp electrical activity of the observers while they performed the task. We used sintered Ag/AgCl electrodes mounted in a 62-electrode cap (Easy-Cap) at scalp positions including the standard 10–20 system positions along with intermediate positions and an additional row of low occipital electrodes. Linked mastoids served as initial common reference and electrode AFz as the ground. Vertical electro-oculogram (vEOG) was bipolarly registered above and below the dominant eye and the horizontal electro-oculogram (hEOG) at the outer canthi of both eyes. Electrode impedance was maintained below 10 kΩ throughout recording. Electrical activity was continuously sampled at 1,024 Hz. Analysis epochs were generated off-line, beginning 500 ms prior to stimulus onset and lasting for 1,500 ms in total. We rejected EEG and EOG artefacts using a [−30 µV; +30 µV] deviation threshold over 200 ms intervals on all electrodes. The EOG rejection procedure rejected rotations of the eyeball from 0.9 deg inward to 1.5 deg downward of visual angle—the stimulus spanned 5.36°×3.7° of visual angle on the screen. Artifact-free trials were sorted using EEProbe (ANT) software, narrow-band notch filtered at 49–51 Hz, and re-referenced to average reference.

### Computation: Mutual Information

In Information Theory [28],[29], Mutual Information *MI*(*X*;*Y* ) between random variables *X* and *Y* measures their mutual dependence. When logarithms to the base 2 are used in Equation 1, the unit of mutual information is expressed in bits.
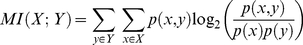
(1)


The critical term is *p*(*x*,*y*), the joint probabilities between *X* and Y. When the variables are independent, the logarithm term in Equation 1 becomes 0 and *MI*(*X*;*Y* ) = 0. In contrast, when *X* and *Y* are dependent *MI*(*X*;*Y* ) returns a value in bits that quantifies the mutual dependence between *X* and *Y*. Derived from the measure of uncertainty of a random variable *X* expressed in Equation 2 and the conditional uncertainty of two random variables *X* and *Y* (Equation 3),
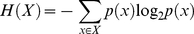
(2)

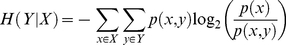
(3)


Mutual Information measures how much bits of information *X* and *Y* share. It quantifies the reduction of uncertainty about one variable that our knowledge of the other variable induces (Equation 4),
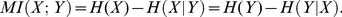
(4)


Here, we use Mutual Information to measure the mutual dependence between the sampling of input visual information from faces and the oscillatory brain responses to these samples and between the same input information and behavior (see Figure S2 for an overall illustration of our framework; see Figure S3 for a detailed development of the computations between face pixels and correct versus incorrect behavioral responses). For all measures of MI, we used the direct method with quadratic extrapolation for bias correction [22]. We quantized data into four equi-populated bins, a distribution that maximizes response entropy [22]. Results were qualitatively similar for a larger number of bins (tested in the range of 4 to 16). Below, we provide details for the computation of mutual information with behavioural and EEG responses, including number of trials taken into consideration for the MI computations and the determination of statistical thresholds of mutual information.

### Behavioral Mutual Information, *MI*(Pixel; Behavior)

On each of the 21,000 trials of a categorization task, the randomly located Gaussian apertures make up a three-dimensional mask that reveals a sparse face. Observers will tend to be correct when this sampled SF information is diagnostic for the categorization of the considered expression. To identify the face features used for each facial expression categorization, we computed mutual information, per observer, between the grey levels of each face pixels and a random sample of correct matching the number of incorrect trials (i.e., on average 5,250 correct trials and 5,250 incorrect trials). For each expression, we then averaged mutual information values across all six observers, independently for each pixel. To establish statistical thresholds, we repeated the computations 500 times for each pixel, after randomly shuffling the order of response—to disrupt the association between pixel values and categorization responses. For each of the 500 computations, we selected the maximum mutual information value across all pixels. We then chose as statistical threshold the 99th percentile of the distribution of maxima. This maximum statistic implements a correction for multiple comparisons because the permutation provides the null distribution of the maximum statistical value across all considered dimensions [30]. Behavioral mutual information is reported as the top row of faces in Figure 1.

### EEG Mutual Information

Here, we examined two different measures: *MI*(EEG Response; Behavior) and *MI*(Pixel; EEG Response). *MI*(EEG Response; Behavior) computed, for each electrode, subject, and expression, the mutual information between correct and incorrect trials and the power, phase, and phase&power of the Time × Frequency EEG signal. For this computation, we used the same number of trials as for Behavior MI (i.e., on average 5,250 correct trials and 5,250 incorrect trials). As with behavior, for each electrode and type of EEG measurement, we averaged the mutual information values across subjects and expression. To establish statistical thresholds, we repeated the computations 500 times, permuting the trial order of the EEG Time × Frequency values and identified the 500 maxima each time across the entire Time × Frequency space. We identified the statistical threshold as the 99th percentile of the distribution of maxima (see Figure 2).

*MI*(Pixel; Phase&Power) computed, for each subject, expression, and face pixel (down-sampled to 38×24 pixel maps), the mutual information between the distribution of each face pixel grey-level value and the most informative of the brain responses, phase&power Time × Frequency responses, for correct trials only. That is, an average of 15,750 trials per subject. To establish statistical thresholds, given the magnitude of the computation, we computed *z* scores using the pre-stimulus presentation baseline (from −500 to 0 ms) to estimate mean and standard deviation. In Figure 3, .01 bits of mutual information correspond to a *z* score of 55.97, so all mutual information values this number of bits (see the level marked with an asterisk in Figure 3) are well above an uncorrected threshold of .0000001 (itself associated with a *z* score of 5).

Figure 2 indicated two clusters of maximal *MI* in all three measures (Power, Phase, and Phase&Power) at a latency of 140–250 ms in two frequency bands (4 Hz and 12–14 Hz). We averaged the *MI* measures, for each cluster, electrode, and subject, and subjected these *MI* averages to a two-way ANOVA with factors electrode (P7, P8, Pz, and Oz) and measure (Power, Phase, and Phase&Power). Both clusters revealed a significant main effect of electrode (*F*(1, 3) = 8.38, *p*<0.001 for 4 Hz and *F*(1, 3) = 79.34, *p*<0.001 for 12–14 Hz) and measure (*F*(1, 2) = 44.24, *p*<0.001 for 4 Hz and *F*(1, 2) = 104.77, *p*<0.001 for 12–14 Hz). Post hoc *t* test confirmed that *MI*(Phase&Power) is significantly higher than *MI*(Phase) (*p* = 0.013), which itself is significantly higher than *MI*(Power) (*p* = 0.003).

## Supporting Information

Figure S1 Illustration of the bubbles sampling procedure. The original stimulus is decomposed into five non-overlapping bands of Spatial Frequencies (SF) of one octave each (120–60; 60–30; 30–15; 15–7.5; 7.5–3.8 cycles per face). We sampled information from each SF band using a mask punctured with Gaussian apertures. These were randomly positioned trial by trial to approximate a uniform sampling distribution of all face regions across trials. We adjusted the size of the apertures for each SF band so as to maintain constant the total area of the face revealed across trials (standard deviations of the bubbles were.36, .7, 1.4, 2.9, and 5.1 cycles/deg of visual angle from fine to coarse). We calibrated the sampling density (i.e., the number of bubbles) on a trial-per-trial basis to maintain a 75% correct categorization performance independently for each expression. The stimulus presented on each trial comprised information from each SF band summed together.
(TIF)

Figure S2 Mutual Information (*MI*) Framework. Pixel. Reduced 38 × 24 pixels space used for analysis (see Figure S1 for a full description of the information sampling used in the actual experiment). *EEG response*. On each trial, we recorded the observer's EEG response. With a size 5 Morlet wavelet, we performed a Time × Frequency decomposition (with a 7.8 ms time step between −500 to 500 ms around stimulus onset and with a 2 Hz step between 4 and 96 Hz). *Behavior* . On each of the 3000 trials per expression (illustrated for “happy”), we recorded the observer's correct versus incorrect responses to the sampled information. *Computation of MI*. Across the 3,000 trials per expression, for each pixel we summed the Gaussian apertures across spatial frequency bands and collected the distributions of resulting grey-level values associated with correct and incorrect responses. We then computed *MI* between the pixel values reflecting the Gaussian apertures and correct versus incorrect responses, *MI*(Pixel; Behavior). We also computed *MI* between behavior and the EEG response, *MI*(EEG Response; Behavior), independently for power, phase, and the conjunction of phase&power. Finally, we computed *MI* between the pixels values and the EEG response, *MI*(EEG Response; Behavior).
(TIF)

Figure S3 Detailed Illustration of the Computation of *MI*(Pixel; Behavior). For one observer, expression “happy,” we provide the full computation of mutual information using two face pixels (P1 and P2) and an equal number of correct (c) and incorrect (i) categorization responses. Note that if the computation had been between face pixels and EEG parameters, we would have had four rows (one per bin of, e.g., amplitude or phase) in the matrix of joint probabilities, not two (for correct and incorrect).
(TIF)

## Abbreviations

CAFÉ: California Facial Expressions
ERP: event related potentials
cortical oscillations
neural coding
FACS: Facial Action Coding System
hEOG: horizontal electro-oculogram
*MI*: Mutual Information
SF: spatial frequency
vEOG: vertical electro-oculogram
