# Supplementary figures and images for: Cracking the Code of Oscillatory Activity
Source: PLoS Biol. 2011 May 17;9(5):e1001064. doi: 10.1371/journal.pbio.1001064 (PMC3096604; doi:10.1371/journal.pbio.1001064)

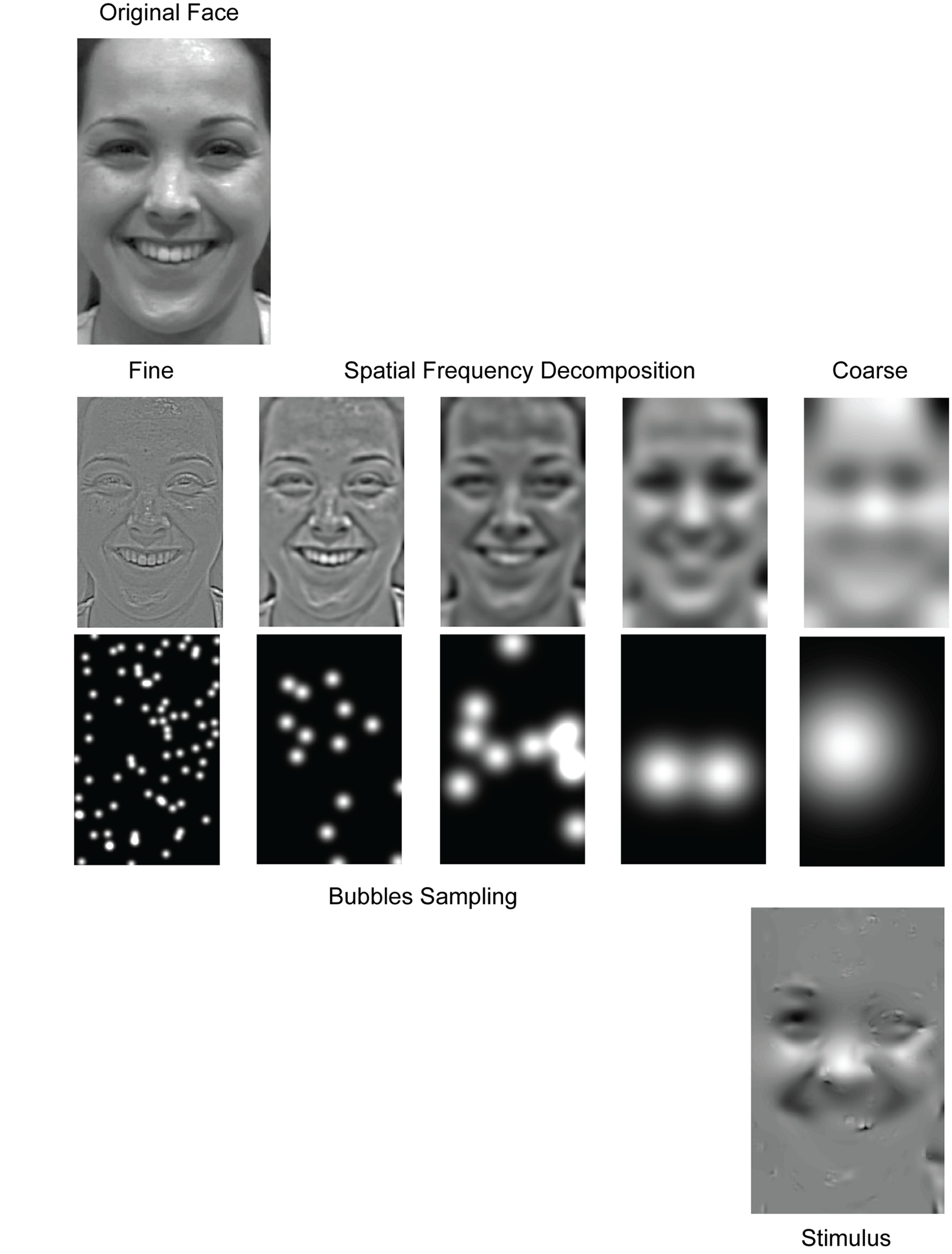

Supplement: Figure S1 — Illustration of the bubbles sampling procedure. The original stimulus is decomposed into five non-overlapping bands of Spatial Frequencies (SF) of one octave each (120–60; 60–30; 30–15; 15–7.5; 7.5–3.8 cycles per face). We sampled information from each SF band using a mask punctured with Gaussian apertures. These were randomly positioned trial by trial to approximate a uniform sampling distribution of all face regions across trials. We adjusted the size of the apertures for each SF band so as to maintain constant the total area of the face revealed across trials (standard deviations of the bubbles were.36, .7, 1.4, 2.9, and 5.1 cycles/deg of visual angle from fine to coarse). We calibrated the sampling density (i.e., the number of bubbles) on a trial-per-trial basis to maintain a 75% correct categorization performance independently for each expression. The stimulus presented on each trial comprised information from each SF band summed together. (TIF) [file pbio.1001064.s001.tif]

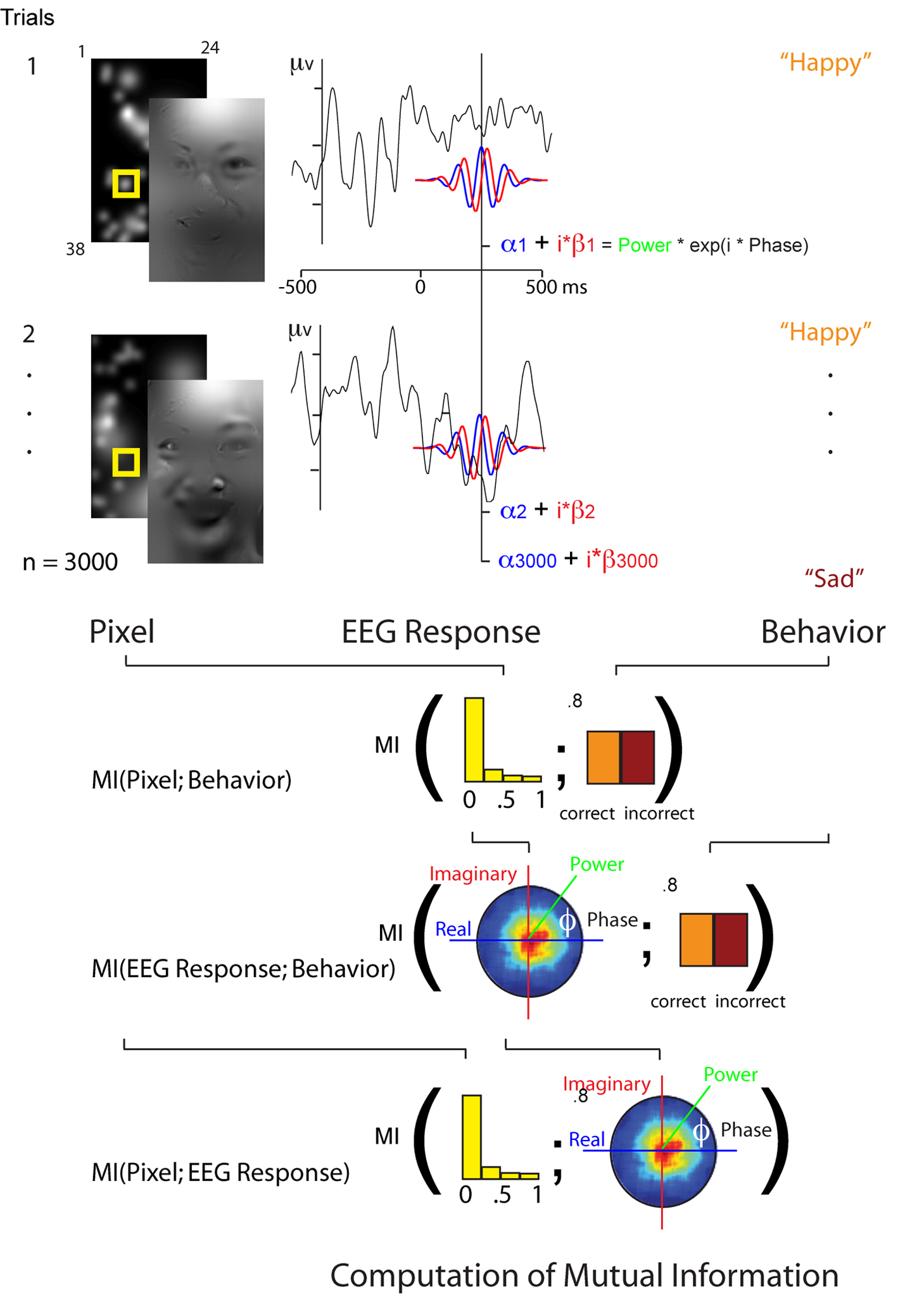

Supplement: Figure S2 — Mutual Information (MI) Framework. Pixel. Reduced 38 × 24 pixels space used for analysis (see Figure S1 for a full description of the information sampling used in the actual experiment). EEG response. On each trial, we recorded the observer's EEG response. With a size 5 Morlet wavelet, we performed a Time × Frequency decomposition (with a 7.8 ms time step between −500 to 500 ms around stimulus onset and with a 2 Hz step between 4 and 96 Hz). Behavior . On each of the 3000 trials per expression (illustrated for “happy”), we recorded the observer's correct versus incorrect responses to the sampled information. Computation of MI. Across the 3,000 trials per expression, for each pixel we summed the Gaussian apertures across spatial frequency bands and collected the distributions of resulting grey-level values associated with correct and incorrect responses. We then computed MI between the pixel values reflecting the Gaussian apertures and correct versus incorrect responses, MI(Pixel; Behavior). We also computed MI between behavior and the EEG response, MI(EEG Response; Behavior), independently for power, phase, and the conjunction of phase&power. Finally, we computed MI between the pixels values and the EEG response, MI(EEG Response; Behavior). (TIF) [file pbio.1001064.s002.tif]

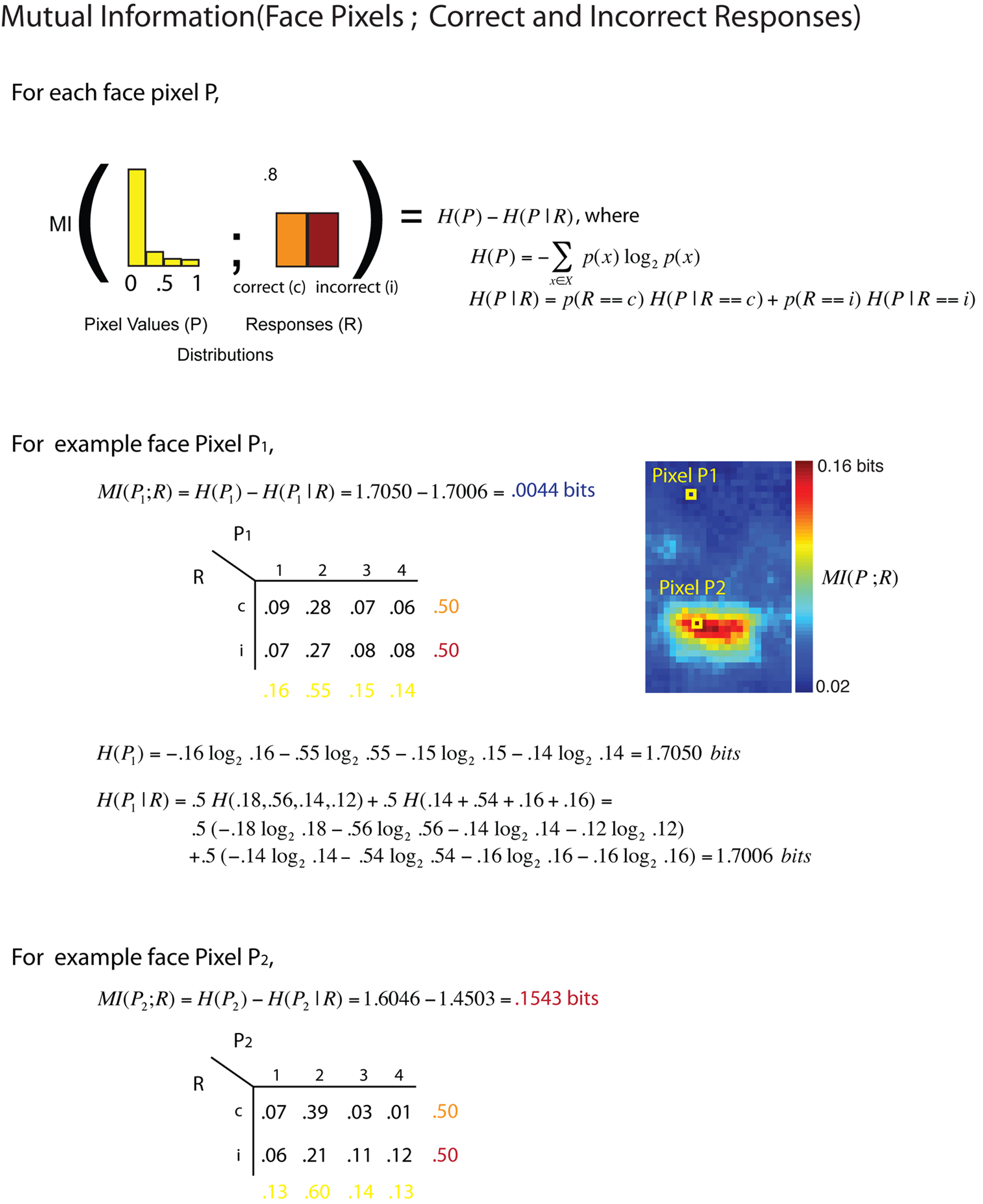

Supplement: Figure S3 — Detailed Illustration of the Computation of MI(Pixel; Behavior). For one observer, expression “happy,” we provide the full computation of mutual information using two face pixels (P1 and P2) and an equal number of correct (c) and incorrect (i) categorization responses. Note that if the computation had been between face pixels and EEG parameters, we would have had four rows (one per bin of, e.g., amplitude or phase) in the matrix of joint probabilities, not two (for correct and incorrect). (TIF) [file pbio.1001064.s003.tif]
